# PATZ1 Is Overexpressed in Pediatric Glial Tumors and Correlates with Worse Event-Free Survival in High-grade Gliomas

**DOI:** 10.3390/cancers11101537

**Published:** 2019-10-11

**Authors:** Annalisa Passariello, Maria Elena Errico, Vittoria Donofrio, Manuela Maestrini, Alia Zerbato, Laura Cerchia, Maria Capasso, Mario Capasso, Monica Fedele

**Affiliations:** 1 Department of Pediatric Oncology, Santobono-Pausilipon Hospital, 80123 Naples, Italy; annalisapassariello75@gmail.com (A.P.); mariellacapasso1969@gmail.com (M.C.); 2 Department of Translational Medical Science Pediatric Section, University of Naples Federico II, 80131 Naples, Italy; manuela91na@gmail.com (M.M.); alia.zerbato@gmail.com (A.Z.); 3 Pathology Unit, Santobono-Pausilipon Hospital, 80123 Naples, Italy; mariaelenaerrico@virgilio.it (M.E.E.); vittoriadono@gmail.com (V.D.); 4 National Research Council (CNR), Institute of Experimental Endocrinology and Oncology “G. Salvatore” (IEOS), 80145 Naples, Italy; cerchia@unina.it; 5 CEINGE Biotecnologie avanzate, 80145 Naples, Italy; mario.capasso@unina.it; 6 Department of Molecular Medicine and Medical Biotechnologies, University of Naples Federico II, 80131 Naples, Italy

**Keywords:** pediatric brain tumors, glioma, PATZ1, prognosis

## Abstract

Glial tumors are the leading cause of cancer-related death and morbidity in children. Their diagnosis, mainly based on clinical and histopathological factors, is particularly challenging because of their high molecular heterogeneity. Thus, tumors with identical histotypes could result in variable biological behaviors and prognoses. The PATZ1 gene has been recently shown to be expressed in adult gliomas, including glioblastomas, where it correlates with the proneural subtype and with a better prognosis. Here, we analyzed the expression of PATZ1 in pediatric gliomas, first at mRNA level in a public database, and then at protein level, by immunohistochemistry, in a cohort of 52 glial brain tumors from young patients aged from 6 months to 16 years. As for adult tumors, we show that PATZ1 is enriched in glial tumors compared to the normal brain, where it correlates positively and negatively with a proneural and mesenchymal signature, respectively. Moreover, we show that PATZ1 is expressed at variable levels in our cohort of tumors. Higher expression was detected in high-grade than low-grade gliomas, suggesting a correlation with the malignancy. Among high-grade gliomas, higher levels of PATZ1 have consistently been found to correlate with worse event-free survival. Therefore, our study may imply new diagnostic opportunities for pediatric gliomas.

## 1. Introduction

Pediatric brain tumors (PBT) account for about 25% of childhood neoplasms and are not only the second most common pediatric cancer, ranking immediately behind leukemia, but also have the highest mortality among pediatric cancers [1]. Among PBTs, glial tumors represent the most common malignant tumors in children, and patients with high-grade gliomas have a very dismal prognosis, with a median survival rate of <1 year, pointing to an urgent need for alternative therapeutic approaches [2]. One of the major challenges in the fight against gliomas is the identification of new molecular data and novel targets responsible for tumor recurrence and progression. Traditionally, pediatric glial tumors have been grouped into low-grade (pLGG) (grade 1–2) and high-grade (pHGG) gliomas (grade 3–4), identified only by histologic parameters: Pilocytic Astrocytoma (G1), Diffuse Astrocytoma (G2), Anaplastic Astrocytoma (G3), and Glioblastoma (GBM) (G4). pLGG are the most common gliomas in the pediatric population. Their clinical signs and symptoms are attributed to mass effect, such as the obstructive hydrocephalus, or focal neurologic deficits. The prognosis generally depends on the site, as well as the age of the patient [3,4]. Conversely, pHGG represents 20% of the PBT [5], is mostly characterized by nuclear atypia, mitotic activity, vascular proliferation, and necrosis, and has a poor prognosis [6]. Recent works in PBT have demonstrated key differences between these tumors compared with adult counterparts [7]. Indeed, the 2016 World Health Organization (WHO) classification of PBT, in defining specific entities, added genetic alterations and molecular findings to the morphologic criteria [8]. Molecularly, pediatric gliomas are characterized by gene amplifications, deletions, and other types of mutations, distinct from the adult counterpart; the most common alterations in pLGG are BRAF mutations, followed by FGFR1 alterations; in pHGG, TP53 and H3F3A mutations dominate the genomic landscape. In particular, mutations at K27 and G34 amino acid residues of the H3F3A gene are the most significant findings in pHGG, whereas they are rare in adult HGG. The identification of these alterations is important for the therapeutic plan, as the presence of the K27M variant is the defining event for the newly recognized WHO entity, termed Diffuse Midline Glioma H3 K27M-mutant, WHO Grade IV, associated with a poor prognosis regardless of histological grade [7].

The POZ/BTB and AT-Hook-Containing Zinc Finger Protein 1 (PATZ1) gene, which maps onto the human chromosome 22q12 [9,10], is a member of the POK [Poxviruses and Zinc-finger (POZ) and Krüppel] family that includes many transcription factors sharing important roles in development, cell proliferation, senescence, and apoptosis [11]. The role of PATZ1 in human cancer regulation is debated, as it works sometimes as a tumor suppressor and sometimes as an oncogene, depending on the cellular context [12]. A potential oncogenic function for PATZ1 has been described in colorectal cancer [13], while it acts as a tumor suppressor in the lung [14] and thyroid [15]. Furthermore, the expression of PATZ1 has been suggested as a prognostic factor for serous ovarian carcinoma [16], diffuse large B-cell lymphomas [17], and renal cell carcinoma [18].

More recently, PATZ1 was revealed to be a potential prognostic marker for glioblastoma in adult patients. Although PATZ1 is generally overexpressed in these tumors compared with the normal brain, its expression levels can predict patients’ survival rates: high expression levels of PATZ1, associated with the proneural subtype, have been found to correlate with a better prognosis, while low levels, associated with the mesenchymal subtype, have correlated with the worst outcomes [19]. Consistently, PATZ1 expression, which has been found in the stem cell compartment of the tumor, is higher in glioma-initiating stem cells (GSCs) growing as spheres (likely proneural) than in GSCs growing as adherent cells (likely mesenchymal). This observation, together with the microscopic demonstration that PATZ1 was expressed in the Nestin^+^ cell subpopulation, reveals why the proneural subtype of GBM resists standard therapies [19].

As far as pediatric gliomas are concerned, comprehensive genomic profiling has identified rearrangement of the PATZ1 gene with the Ewing Sarcoma-related gene, EWSR1 in a pHGG [5]. A similar rearrangement was also found in glioneural tumors, suggesting it might define a new type of glioneuronal tumor distinct from gangliogliomas [20,21].

The purpose of our study was to analyze the expression of PATZ1 in childhood gliomas and correlate it with the clinicopathological features, with the final aim of opening up a novel diagnostic and potential therapeutic option for children with this challenging malignancy.

## 2. Results

### 2.1. PATZ1 Gene Expression Is Enriched in Pediatric Glial Tumors and Associated with a Proneural Signature

**Figure 1:**
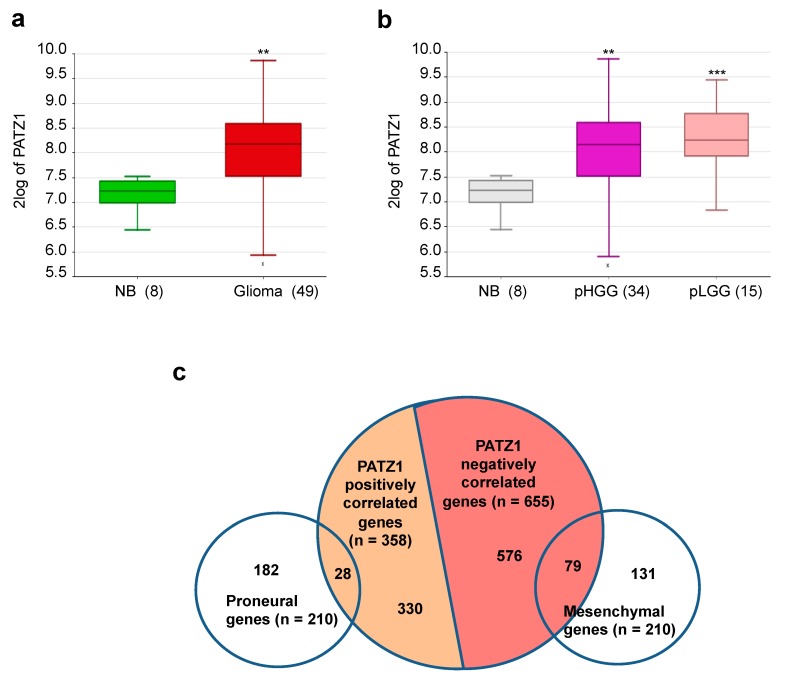
In silico analysis of PATZ1 expression in pediatric glioma tissues. (**a**) Box plot comparing PATZ1 expression in normal brain (NB) and pediatric gliomas, including both low- (pLGG) and high-grade (pHGG) tumors (GSE50161). The data were analyzed by one-way analysis of variance (ANOVA) through the R2 web platform. The number of tissues is indicated in brackets. ** *p* < 0.01 versus NB. (**b**) Box plot showing PATZ1 expression in the two different subtypes compared with normal control. No differences were observed between pLGG and pHGG. The number of tissues is indicated in brackets. ** *p* < 0.01; *** *p* < 0.001 versus NB. (**c**) Schematic representation of the overlapping between PATZ1-correlated genes in pediatric gliomas and either proneural or mesenchymal genes of the adult glioblastoma signatures described by Verhaak et al. [23].

To evaluate the expression of PATZ1 in pediatric gliomas, we initially analyzed public databases by using the R2: Genomic Analysis and Visualization platform (http://r2.amc.nl), screening a microarray dataset of pediatric brain samples (GSE50161), including eight normal cerebral cortex tissues obtained from autopsy or epilepsy surgery and 49 glial tumors (15 pLGG and 34 pHGG) [22] that we used in our study. Similar to adult glial tumors, pediatric glial tumors showed a significant increase of PATZ1 expression compared to normal brain tissues (*p* = 1.33 × 10^−3^ (Figure 1a). However, there was no significant difference in PATZ1 expression between pLGG and pHGG (Figure 1b). Interestingly, among pHGG, as for adult GBM [19], PATZ1 expression appeared to correlate positively with the proneural, and negatively with the mesenchymal signatures. Indeed, 28 proneural genes and 79 mesenchymal genes, as defined by Verhaak et al. [23], were significantly correlated either positively or negatively with PATZ1 expression, respectively (Figure 1c and Table 1).

### 2.2. PATZ1 Protein Expression Is Associated with Tumor State in Pediatric Glioma Tissue and More Frequently Highly Expressed in pHGG than pLGG

**Figure 2:**
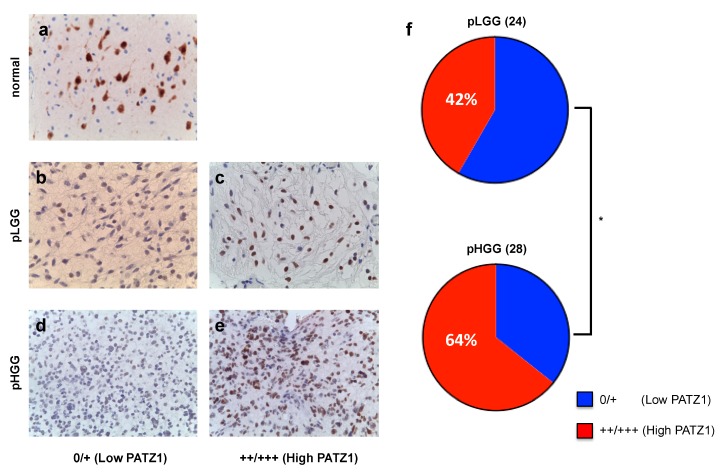
Immunoreactivity score in pediatric gliomas stained for PATZ1. (**a**) Representative perilesional normal cortex: only neurons stain positively, while glial cells are negative. (**b**) Representative pLGG scored low (≤10% PATZ1-positive cells). (**c**) Representative pLGG scored high (>10% PATZ1-positive cells). (**d**) Representative pHGG scored low. (**e**) Representative pHGG scored high. (**f**) Fraction of total PATZ1 scores in pLGG and pHGG. Percentage of high PATZ1 expression is indicated. Discrepancy was significant according to the binomial test. * *p* < 0.05.

To further investigate PATZ1 expression in pediatric glial tumors and correlate it with clinicopathological features, we collected tumor samples, including 24 pLGG and 28 pHGG from 52 young patients aged from 6 months to 16 years, and analyzed PATZ1 expression by immunohistochemistry (IHC) using a polyclonal antibody able to recognize all PATZ1 isoforms [19]. Perilesional normal cortex, used as the control, showed a nuclear positivity only in neurons, whereas glial cells were totally negative for PATZ1 expression (Figure 2a). Conversely, most of both pLGG (54%) and pHGG (79%) resulted positive for PATZ1 expression with different percentages of positivity, ranging from absent to 90% (Table 2 and Figure 2, respectively). Based on the percentage of PATZ1 positive cells, we grouped the tumors into two categories: low PATZ1 (0/+) and high PATZ1 (++/+++) (Figure 2 and Table 3, respectively). Then, we assessed the correlations between PATZ1 expression levels and the clinicopathological features to explore the potential clinical significance of PATZ1 in pediatric gliomas. As shown in Figure 2b and Table 3, high PATZ1 was associated with the tumor grade, with pHGG showing higher levels of PATZ1 compared to pLGG. Statistically, this association was significant (*p* = 0.022) when considering the relative frequencies and applying the binomial test (Figure 2b), but it was shown to have just a trend to significance (*p* = 0.088) by applying Fisher’s exact test (Table 3). A correlation trend (*p* = 0.076) was also present between metastatic cases and the high PATZ1 group. Indeed, 6 out of 7 patients with metastases (86%) showed high expression of PATZ1 in the glioma sample (Table 3). Further analyses by expanding the number of patients are necessary to confirm these clinical associations.

### 2.3. High PATZ1 Expression Correlates with Worse Event-Free Survival in pHGG

**Figure 3:**
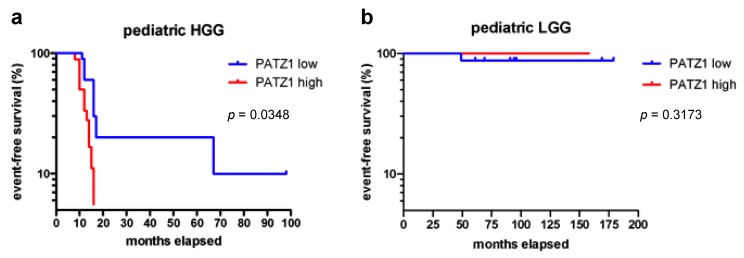
PATZ1 expression found to correlate with worse event-free survival in pHGG. Event-free Kaplan-Meier survival curves of our local cohort of (**a**) 28 pHGG and (**b**) 24 pLGG patients stratified by protein levels of PATZ1, as indicated. pHGG patients with high PATZ1 levels had worse survival rates, as assessed by the log-rank test (*p* < 0.05).

We next analyzed the correlation between PATZ1 expression and patients’ clinical outcomes by survival curves and log rank test. As shown in Figure 3a, PATZ1 expression levels stratified pHGG patients into two subgroups with different event-free survival (EFS) rates, where patients with a high PATZ1 score (++/+++) had worse EFS than patients with a low PATZ1 score (0/+) (HR = 0.492, 95% CI = 0.155–0.9335, *p* = 0.0348). The median EFS for 18 young patients with high PATZ1 was 11 months, in contrast to 16 months for 10 young patients with low or absent PATZ1. Conversely, no differences were observed in EFS of pLGG (Figure 3b) or overall survival (OS) of both pHGG and pLGG.

## 3. Discussion

The genetic basis of childhood brain tumors has been deeply characterized in the last 5–10 years, helping in the identification of different subsets of tumors that have been recognized by the WHO [4], thus allowing specific therapeutic approaches for many of them. However, for some of them, such as pHGGs, translation of this knowledge to therapeutics remains a work in progress. It recently emerged that pediatric HGGs are molecularly different from adult HGGs, therefore implying that different targeted strategies are needed [8]. Specifically, the clinically relevant subtypes of GBM found in adults by Verhaak et al. [23] are not as well defined in pediatric GBM, but genetic profiling revealed *PDGFRα,* a key gene of the adult proneural subtype, as the predominant target of focal amplification in pHGG [24,25]. Here, we reported that the PATZ1 gene, which is highly expressed in adult GBMs and associated with the proneural subtype [19], is also highly expressed in a large subset of pediatric glial tumors. Interestingly, even in pHGG, PATZ1 expression is associated positively with proneural genes and negatively with mesenchymal genes of the adult neoplasms, thus suggesting a common mechanism of action of PATZ1 in both adult and childhood glial tumors, likely based on the counteraction of the proneural-to-mesenchymal transition (PMT) [19]. In high-grade gliomas of adult patients, while the proneural signature is associated with a better prognosis, the mesenchymal one is associated with a poor clinical outcome, and a switch from the proneural to mesenchymal phenotype can occur upon recurrence [26]. Accordingly, in these tumors, PATZ1 is associated with better patient survival rates (OS and EFS) [19]. Conversely, we showed in this paper that in pHGG, high PATZ1 levels are not associated with OS, but instead correlated with worse EFS, confirming that gliomas from adult and childhood patients are two different cancer entities [25,27].

Differently from OS, which measures the proportion of patients still alive at a specified time after diagnosis, EFS measures the proportion of people, among those treated, who remained alive and free of disease at a specified time after treatment. Therefore, the correlation between high PATZ1 and worse EFS may imply a crucial role for PATZ1 in causing tumor recurrence after treatment. This is consistent with a previous in vitro study showing that PATZ1 silencing in glioma cells enhanced their sensitivity to chemotherapeutic agents [28]. One of the key drivers of chemoresistance and relapse are cancer stem cells, due to their intrinsic resistance to therapy and tumor-initiating capacity [29]. PATZ1 is known to localize in the stemness tumor compartment, where it can contribute to the maintenance of the glioma stem cells [19], similarly to what has been established in mouse embryonic stem cells [30]. This could explain why its enhanced expression is correlated with major treatment resistance. Indeed, differently from adult GBM, in which the intrinsic chemoresistance has been related to PMT, very little is known on the role of PMT in pHGG. It has been reported that major differences can be found in the expression of certain key components of the mesenchymal phenotype. For instance, the expression of EGFR—a common PMT inducer—was much higher in adult GBM compared to pHGG, suggesting a more limited role of this signaling in pediatric glioma than in the adult counterpart [31]. In this light, the selected genes, directly correlated with PATZ1 expression and belonging to the proneural signature, could be potential targetable molecules for new therapeutic approaches against pediatric gliomas. Further studies on the specific role of PATZ1 and its related genes in glioma stem cells will clarify this issue. Overall, our data provide new insights for therapeutic stratification of young patients with HGG into risk groups, suggesting a new molecular target for therapy.

## 4. Materials and Methods

### 4.1. Data Mining

For PATZ1 mRNA expression analysis and correlation with molecular and clinical phenotypes, the Genomics Analysis and Visualization platform (http://r2.amc.nl) was used on gene expression profiles generated from 130 mixed pediatric brain tumors and normal brain samples (GSE50161) through Affymetrix HG-U133plus2 chips (Platform GPL570) [22], by selecting a subset including 15 pilocytic astrocytomas, 34 GBMs, and eight normal brain cortex samples obtained from autopsy or epilepsy surgery.

### 4.2. Patients

Our local study population consisted of 52 patients—28 males and 24 females, aged between 6 and 192 months (16 years), with tumors localized in the optical tract (19 cases), brain stem (9 cases), cortex (10 cases), thalamus (eight cases), cerebral ventricles (three cases), and posterior cranial fossa (three cases). The cases, diagnosed and/or revised according to the 2016 WHO classification, comprised 24 pLGG (all Pylocytic astrocytomas, including two pilomixoid) and 28 pHGG (26 GBMs, two anaplastic astrocytomas).

Six patients (#2, #9, #13, #15, #26, #40) had Neurofibromatosis 1 (NF1) that is frequently associated with the appearance of pilocytic astrocytoma.

The tumor onset symptoms were intracranial hypertension in 28 patients (9 pLGG, 19 pHGG), nystagmus and visual deficits in 10 patients (8 pLGG, 2 p HGG), ataxia in one patient with pHGG, epilepsy in two patients (1 pLGG, 1 pHGG), language disorders, such as dysarthria or aphasia, in four patients (1 pLGG, 3 pHGG), asymmetric paresis in three patients (2 pLGG, 1 pHGG), diencephalic syndrome in two patients with pLGG, precocious puberty in one patient with pLGG, and in one patient the diagnosis was achieved accidentally.

Regarding the treatment, the patients with pLGG received the SIOP LGG 2004 protocol [32] and 11 patients (#1, #2, #3, #5, #6, #7, #11, #12, #17, #19, #20) also received radiotherapy. The patients with pHGG received the SNC HR protocol [33,34], and 18 patients (#25, #28, #29, #30, #31, #32, #33, #35, #36, #37, #40, #41, #43, #44, #47, #48, #49, #52) also received radiotherapy. The toxicity during the treatments involved 13 patients with pLGG (#1, #2, #3, #5, #7, #8, #9, #10, #13, #15, #18, #20, #24), where six patients in particular (#7, #9, #13, #15, #18, #24) were allergic to carboplatin and presented with peripheral neuropathies and erythematous lesions; five patients (#1, #2, #5, #8, #20) had hematologic toxicity, and two patients (#3, #10) had audiological toxicity. Fourteen patients (#1, #2, #3, #4, #6, #7, #8, #10, #13, #15, #19, #20, #21, #24) had hormonal complications after treatment, and two patients (#17, #41) had neurological complications after surgical treatment. Of all the patients, three with pLGG (#6, #18, #20) had four recurrences, and one (#21) had six recurrences. Seven patients (#1, #25, #26, #27, #28, #29, #30) had metastasis, mostly localized in the spine and leptomeninges.

Of all the patients we studied, 29 died (two with pLGG and 27 with pHGG) from tumor progression, two were transferred to other hospitals, and 21 are currently in follow-up. Informed consent for the scientific use of biological material was obtained from all patients, and the study protocol was approved by the Ethics Committee of the Medical School of the University of Naples “Federico II”, (protocol number 238/17, released on 10/07/2018).

### 4.3. Histopathological Analysis and Immunohistochemistry

Surgically removed tissues were fixed by immersion in 10% buffered formalin for at least 24 h and then embedded in paraffin using standard procedures at the Pathology Unit of Santobono-Pausilipon Hospital of Naples. Sections (4 μm) were stained with hematoxylin and eosin using standard histologic techniques, or processed for the immunohistochemistry. Diagnoses were made in accordance with the 2016 WHO classification of central nervous system tumors [4]. All tumors were classified based on histological parameters and graded as pLGG and pHGG. For immunohistochemical analysis, 4 μm formalin-fixed paraffin-embedded sections of each tumor were incubated with antibody anti-PATZ1 (custom polyclonal rabbit, R1P1, Primm Biotech, Milan, Italy [19]; 1: 500 dilution) raised against a peptide in the N-terminal region of the human PATZ1 protein (aa 1–276) (Figure 2). The endogenous peroxidase activity was blocked by incubating the sections with Peroxidase-Blocking Reagent (Agilent DAKO, Santa Clara, CA, USA) for 10 min at room temperature, followed by two washes (2 min/each) in Tris Buffered Solution (TBS). The slides were incubated with about 200 μL of primary antibodies for 1 h at room temperature, followed by two washes in TBS buffer (2 min/each), secondary antibody (HRP, Agilent DAKO) for 30 min at room temperature, two washes in TBS/Tween (2 min/each), and then visualized using a 3,3′-diaminobenzidine (DAB + Chromogen, Agilent DAKO). The negative controls were obtained with omission of the primary antibody, while the perilesional normal cerebral cortex was evaluated as a normal control. The assessment of PATZ1 staining was based on the percentage of positive cells: no signal was set as “0”, ≤10% as “+”, >10%–<50% as “++” and ≥50% as “+++”. We defined high expression of PATZ1 as >10% of cells staining positive, and low expression as ≤10% of cells staining positive.

### 4.4. Statistical Analysis and Kaplan-Meier Survival Curves

Fisher’s exact test was used to evaluate the correlations between the PATZ1 score and the clinical variables (i.e., age, gender, site, grade, metastasis, relapse). The binomial test was used to compare the fraction of the total of two groups of data through the GraphPad Prism 5 software (San Diego, Ca, USA). All correlations with the public dataset were assessed by Pearson’s χ^2^ test or one-way analysis of variance (ANOVA) through the R2 platform (http://r2.amc.nl). Kaplan-Meier survival curves were used to analyze OS and EFS, and statistical significance was assessed by the log-rank test through the GraphPad Prism 5 software. For all statistical analyses, a probability (*p*) value less than 0.05 was considered significant.

## 5. Conclusions

High-grade gliomas, which, for a long time, were considered a single tumor entity, are now accepted to comprise many subsets of tumors that differ in terms of prognosis. In children, they are different from those that arise in adults, but share with the adult GBMs the high incidence of recurrence after therapy, which is then responsible for death. Therefore, a major challenge in the fight against GBM in both adults and children is to hit molecular targets responsible for tumor recurrence. Here, we provided evidence that, differently from adult GBMs, high PATZ1 expression can predict a higher rate of recurrence in pediatric HGG, thus opening up new diagnostic and possibly therapeutic options for children with these challenging tumors.

## Figures and Tables

**Table 1:** Correlations between PATZ1 and the proneural and mesenchymal signature in pediatric glioblastoma (*n* = 34) ^1.^

Proneural Signature			Mesenchymal Signature		
Gene	r ^2^	*p*	Gene	r	*p*
*PAFAH1B3*	0.785	4.5 × 10^−5^	*ITGAM*	−0.832	6.1 × 10^−6^
*LOC81691*	0.745	1.4 × 10^−4^	*PLS3*	−0.817	1.3 × 10^−5^
*CHD7*	0.713	3.7 × 10^−4^	*MAN1A1*	−0.780	5.5 × 10^−5^
*MAP2*	0.673	1.0 × 10^−3^	*CASP4*	−0.776	5.9 × 10^−5^
*HDAC2*	0.663	1.2 × 10^−3^	*S100A13*	−0.773	6.4 × 10^−5^
*MTSS1*	0.639	2.2 × 10^−3^	*PTPRC*	−0.768	7.7 × 10^−5^
*DLL3*	0.633	2.5 × 10^−3^	*AMPD3*	−0.767	7.3 × 10^−5^
*SOX4*	0.630	2.6 × 10^−3^	*ALOX5*	−0.755	1.0 × 10^−4^
*MARCKSL1*	0.630	2.6 × 10^−3^	*VDR*	−0.751	1.2 × 10^−4^
*HN1*	0.624	3.0 × 10^−3^	*TNFRSF11A*	−0.742	1.6 × 10^−4^
*PKMYT1*	0.624	3.0 × 10^−3^	*CASP8*	−0.741	1.6 × 10^−4^
*PODXL2*	0.620	3.2 × 10^−3^	*PLA2G5*	−0.741	1.6 × 10^−4^
*CASK*	0.614	3.6 × 10^−3^	*MFSD1*	−0.737	1.9 × 10^−4^
*DBN1*	0.609	4.1 × 10^−3^	*RUNX2*	−0.732	2.1 × 10^−4^
*GNG4*	0.609	4.1 × 10^−3^	*RAC2*	−0.726	2.7 × 10^−4^
*SEZ6L*	0.608	4.1 × 10^−3^	*CASP1*	−0.726	2.7 × 10^−4^
*SLCO5A1*	0.605	4.4 × 10^−3^	*LY75*	−0.724	2.8 × 10^−4^
*MCM10*	0.602	4.6 × 10^−3^	*CAST*	−0.719	3.3 × 10^−4^
*CSNK1E*	0.598	5.0 × 10^−3^	*IL15*	−0.719	3.4 × 10^−4^
*RAB33A*	0.591	5.8 × 10^−3^	*LY96*	−0.717	3.4 × 10^−4^
*ASCL1*	0.591	5.7 × 10^−3^	*TRIM38*	−0.709	4.2 × 10^−4^
*HMGB3*	0.590	5.8 × 10^−3^	*ARSJ*	−0.706	4.5 × 10^−4^
*SOX11*	0.587	6.1 × 10^−3^	*SQRDL*	−0.697	5.6 × 10^−4^
*AMOTL2*	0.582	6.6 × 10^−3^	*TLR2*	−0.691	6.4 × 10^−4^
*OLIG2*	0.576	7.3 × 10^−3^	*SYPL1*	−0.688	7.0 × 10^−4^
*DPF1*	0.569	8.4 × 10^−3^	*PTRF*	−0.684	7.8 × 10^−4^
*TOPBP1*	0.567	8.6 × 10^−3^	*VAMP5*	−0.680	8.6 × 10^−4^
*CRMP1*	0.566	8.8 × 10^−3^	*ITGB2*	−0.675	9.6 × 10^−4^
			*SCPEP1*	−0.674	9.8 × 10^−4^
			*PTGER4*	−0.672	1.0 × 10^−3^
			*RAB27A*	−0.667	1.2 × 10^−3^
			*TNFRSF1A*	−0.667	1.1 × 10^−3^
			*CD14*	−0.667	1.2 × 10^−3^
			*CHI3L1*	−0.666	1.2 × 10^−3^
			*TRADD*	−0.664	1.2 × 10^−3^
			*IQGAP1*	−0.664	1.2 × 10^−3^
			*LCP2*	−0.660	1.3 × 10^−3^
			*MSR1*	−0.654	1.6 × 10^−3^
			*ARPC1B*	−0.653	1.6 × 10^−3^
			*TGFBR2*	−0.649	1.8 × 10^−3^
			*LAPTM5*	−0.647	1.8 × 10^−3^
			*PHF11*	−0.646	1.9 × 10^−3^
			*NPC2*	−0.643	2.0 × 10^−3^
			*LAIR1*	−0.642	2.0 × 10^−3^
			*CTSB*	−0.641	2.1 × 10^−3^
			*MAFB*	−0.641	2.1 × 10^−3^
			*NCF2*	−0.640	2.1 × 10^−3^
			*ASL*	−0.637	2.3 × 10^−3^
			*ANXA1*	−0.637	2.3 × 10^−3^
			*S100A11*	−0.629	2.6 × 10^−3^
			*WWTR1*	−0.629	2.6 × 10^−3^
			*COL8A2*	−0.626	2.9 × 10^−3^
			*CSTA*	−0.625	2.9 × 10^−3^
			*IL4R*	−0.619	3.3 × 10^−3^
			*STAB1*	−0.617	3.4 × 10^−3^
			*S100A4*	−0.613	3.7 × 10^−3^
			*MGST2*	−0.609	4.1 × 10^−3^
			*P4HA2*	−0.608	4.1 × 10^−3^
			*MVP*	−0.607	4.2 × 10^−3^
			*MFSD1*	−0.601	4.7 × 10^−3^
			*LCP1*	−0.599	5.0 × 10^−3^
			*STAT6*	−0.593	5.5 × 10^−3^
			*HFE*	−0.592	5.7 × 10^−3^
			*PLAUR*	−0.592	5.7 × 10^−3^
			*FCGR2B*	−0.591	5.7 × 10^−3^
			*ANXA4*	−0.589	5.9 × 10^−3^
			*SP100*	−0.588	5.9 × 10^−3^
			*COPZ2*	−0.584	6.4 × 10^−3^
			*THBD*	−0.584	6.4 × 10^−3^
			*FCGR2A*	−0.582	6.6 × 10^−3^
			*CYBRD1*	−0.579	7.0 × 10^−3^
			*LILRB3*	−0.579	7.0 × 10^−3^
			*IL15RA*	−0.576	7.4 × 10^−3^
			*GNA15*	−0.574	7.6 × 10^−3^
			*PTPN6*	−0.572	7.9 × 10^−3^
			*SLC11A1*	−0.571	8.1 × 10^−3^
			*CLCF1*	−0.570	8.2 × 10^−3^
			*SYNGR2*	−0.566	8.9 × 10^−3^
			*DSC2*	−0.560	9.9 × 10^−3^

^1^ GEO dataset GSE50161. ^2^ Correlations were analyzed by Pearson’s χ^2^ test through the R2 platform (http://r2.amc.nl).

**Table 2:** Clinicopathological features and PATZ1 expression of 52 pediatric gliomas.^1^

Patient	Gender ^2^	Age (months)	Site ^3^	Subtype	Metastases	PATZ1
1	M	49	ch	LGG	NO	50%
2	F	38	ch	LGG	NO	5%
3	F	6	ch	LGG	NO	10%
4	F	102	ch	LGG	NO	5%
5	F	45	ch	LGG	NO	5%
6	F	12	ch	LGG	NO	20%
7	F	93	ch	LGG	NO	20%
8	F	33	ch	LGG	NO	20%
9	F	14	ch	LGG	NO	40%
10	F	88	pv	LGG	NO	60%
11	F	14	ch	LGG	NO	0
12	F	144	pv	LGG	NO	0
13	F	23	ch	LGG	NO	0
14	F	28	ch	LGG	NO	0
15	M	57	ch	LGG	NO	10
16	M	57	ch	LGG	NO	10
17	M	77	ch	LGG	NO	5
18	M	33	ch	LGG	NO	25
19	M	180	pv	LGG	NO	30
20	M	12	ch	LGG	NO	40
21	M	7	ch	LGG	NO	55
22	M	44	pv	LGG	NO	0
23	F	65	co	LGG	NO	0
24	F	9	pv	LGG	NO	0
25	F	171	th	HGG	YES	20
26	F	87	ch	HGG	YES	50
27	F	140	tr	HGG	YES	30
28	F	152	co	HGG	YES	0
29	M	119	tr	HGG	YES	50
30	M	52	th	HGG	YES	20
31	F	109	tr	HGG	NO	30
32	F	121	th	HGG	NO	40
33	F	149	co	HGG	NO	20
34	F	125	co	HGG	NO	40
35	F	143	tr	HGG	NO	80
36	F	164	th	HGG	NO	0
37	M	192	th	HGG	NO	5
38	M	153	tr	HGG	NO	5
39	M	101	co	HGG	NO	5
40	M	76	pv	HGG	NO	5
41	M	89	th	HGG	NO	5
42	M	56	th	HGG	NO	40
43	M	108	tr	HGG	NO	30
44	M	96	co	HGG	NO	40
45	M	139	co	HGG	NO	30
46	M	68	tr	HGG	NO	90
47	M	65	tr	HGG	NO	70
48	M	40	co	HGG	NO	55
49	M	126	co	HGG	NO	0
50	M	91	th	HGG	NO	0
51	M	82	tr	HGG	NO	0
52	M	148	co	HGG	NO	0

^1^ Local cohort analyzed by immunohistochemistry. ^2^ M, male; F, female. ^3^ ch, chiasma; pv, posterior ventricle; co, cortex; ta, thalamus; tr, trunk.

**Table 3:** Correlation between PATZ1 expression and clinicopathological characteristics of pediatric glioma patients (*n* = 52).

Variables	Number	PATZ1 Expression		*p* value ^1^
		High	Low	
**Gender**				
Male	28	16	12	0.407
Female	24	12	12	
**Age (years)**				
≤3	11	5	7	0.674
>3 ≤10	27	16	10	
>10 ≤ 16	14	7	7	
**Grade**				
LGG	24	10	14	0.088
HGG	28	18	10	
**Location**				
Chiasma/Thalamus	27	14	13	0.461
Trunk	9	7	2	
Cortex	10	5	5	
posterior ventricle	6	2	4	
**Metastasis**				
Yes	7	6	1	0.076
No	45	22	23	
**Relapse**				
Yes	20	10	10	0.438
No	32	18	14	

^1^ As assessed by Fisher’s exact test (two sets of data) or linear-to-linear association (more than two sets of data).
